# Reviews of Books

**Published:** 1924-06

**Authors:** 


### REVIEWS OF BOOKS.

### A GARDEN OF HEALING.

**The Romance of the Apothecaries' Garden at Chelsea.** By F. D. Drewitt, M.D., F.R.C.P. Second Edition. (Chapman and Dodd. 5s. net.)

To-day, when we treat all the ills that flesh is heir to by vaccines, inoculations and glandular extracts, there is a tendency to neglect the study of the vegetable *materia medica* which loomed so large in the curative *armamentarium* of our forefathers. Doubtless the curriculum of the medical student is crowded, and it is perhaps vain to regret the passing of the study of plants and their medicinal virtues, but to its close association with medicine the science of botany owes much of its early advances. This is evident from the large number of physicians of past generations who have been notable for their knowledge of the natural history of plants.

When the Apothecaries' Company broke away from that of the Grocers, and in 1632 established themselves in their own Hall at Blackfriars, they attached thereto a small garden for the growth of medicinal herbs. The Civil War, the Plague, and lastly the Great Fire interrupted their plans, and with the rebuilding of their Hall the members of the Company found it advisable to look farther afield for space for a garden in which they could cultivate rare plants and rare seeds brought from overseas. By great good fortune they were able to secure some three and a-half acres in the country Manor of Chelsea, then surrounded by cornfields and pasture lands. For two centuries and a-half the garden has existed on the same spot, and the real romance of its history is now told by Dr. Drewitt, to whom its compilation has clearly been a labour of love. Vast have been the changes the garden has seen since the Stuarts, when the wild bugloss was growing about Piccadilly, belladonna in Islington, and the fratillery lifted its snake's-head flowers in the meadows of Battersea. The reputation of the garden soon spread, and botanists from the Continent came to see the new and strange trees and plants which found a home there. Evelyn, writing in 1685, says:—"I went to see Mr. Watts, keeper of the Apothecaries' Garden at Chelsea, where there is a collection of innumerable rarities of the sort; particularly, besides many rare annuals, the tree bearing Jesuit's Bark, which had done such wonders in Quartan Agues."

But hard times were in store for the Company, and despite the application of taxation upon members for the maintenance of the garden, their means fell short of their plans. It was then that Sir Hans Sloane, to whose interest in science we owe the British Museum, came to their aid. Having purchased the Manor of Chelsea in 1722, he conveyed the garden to the Apothecaries in perpetuity to enable the Company "to support the charge thereof for the manifestation of the power, wisdom and glory of God in the works of Creation." But Sloane conferred another benefit by the introduction of Philip Miller, the author of "The Gardeners' Dictionary," as gardener. It was during his period of service that Linnæus made a special visit to England to see the garden, and was taken to see the yellow blaze of the gorse on Putney Heath. A few years later his pupil Kalm arrived on his botanical expedition to America, followed by Fabricius of Kiel. It was Miller who sent the first packet of cotton seed to the newly established Colony of Georgia. Dr. Drewitt follows no narrow road, but strays into many delightful by-paths of history and botanical lore. He tells how the first bunch of bananas brought to England was exhibited by Thomas Johnson in 1633 in his shop window in Snow Hill; and how the only book ever issued by the Royal College of Physicians was one of seven volumes on birds, by George Edwards, Librarian to the College. Above all, he shows on every page a love of plants that is infectious. Dull indeed must be the reader who does not feel some touch of the same enthusiasm. The sixteen photographic half-tone illustrations of the garden and its charms are admirable.

### REJUVENATION BY ANIMAL GRAFTS.

**Quarante-trois Greffes du Singe a l'Homme.** Par Serge Voronoff. (Paris: Gaston Doin. 20 franc.)

The time would seem to be not far distant when man will enjoy, with the motor-car, the privilege of renewing life by replacing worn-out structures with spare parts. The glands of internal secretion have been found to play an important part, not merely in the reproduction of the species, but in the vitality and viability of the whole body. Less than a generation ago we were taught that old age was the product of certain bacilli in the digestive tract. Now we are asked to believe that old age is the outcome of defective secretions of certain glands among which the gonads play a most important part. This theory is at present being examined along three different lines. There is the Steinach school, which attempts to promote the internal secretions of the male reproductive organs by the simple ligature of their main duct; there is the "extract" school, which seeks to promote rejuvenation by giving the subjects of premature senile decay extracts of testicles and similar organs; and there is finally the "graft" school of which Sergius Voronoff is a distinguished representative. He has already published eleven works on animal grafts, and his present one deals with forty-three grafts from monkey to man carried out since June 12, 1920. These operations were performed for a variety of conditions such as infantalism, testicular insufficiency, neurasthenia, arteriosclerosis, premature old age and general debility.

The author tabulates his results in a precise manner which enables the reader to see at a glance what these grafting operations can achieve. Only in five cases, or 12 per cent., did the operation fail to achieve what was hoped of it. In thirty-six cases, or 88 per cent., good results were obtained physically and mentally, and in twenty-six cases, or 65 per cent., sex virility was restored or increased, in addition to the improvement effected in the patient's mental and general health. A clear account, illustrated by numerous drawings, is given of the author's grafting operation, and he adds interest to his work by publishing photographs of some of his patients before and after the operation. Some of these photographs are dramatically convincing. The reader is first introduced to a feeble, wrinkled old man with heavy eyelids, a dull expression and the characteristic appearance of advanced senility. A few pages further on he is again on show, this time bounding over a lawn with the alert aspect of a Marathon winner and an expression which can best be described as "the glad eye." Yet this man is seventy-seven. It would seem as if we are on the point of penetrating Methuselah's secret, but whether it will add greatly to the happiness of man is another matter.

### THE MENTAL NURSE.

**Addresses to Mental Nurses.** Edited and arranged by Bedford Pierce, M.D., F.R.C.P. (Baillière, Tindall and Cox. 7s. 6d. net.)

In pursuance of the enlightened policy which characterised his administration of the Retreat at York, Dr. Bedford Pierce arranged that special lectures should be given to the nursing staff, and this volume embodies these addresses. They are in addition, of course, to the many lectures which are given annually by members of the medical staff at the Retreat and at other mental hospitals, as part of the training and in preparation for the examinations which are held under the ægis of the Medico-Psychological Association. It might be thought that this would be enough—or more than enough—in the matter of lectures, and that nurses would be unlikely to want anything more of the kind. It may be so, yet experience shows that nurses are avid of lectures. It should be a source of gratification to the public to realise that this is so, especially when so much is being said in disparagement of the work of almost all those who have charge of the insane.

Much less than justice is done to the mental nurse. After more than forty years spent among the insane, Dr. Yellowlees says in his "Address," "The longer I lived among insane people, the more did I realise that their care and treatment is the most difficult work anyone can undertake." Yet Sir George Savage, in the course of his remarks, stated: "The widespread notion that the insane are badly treated in mental hospitals, and that nurses trained in them are naturally rather brutal, leads the relations almost always to ask the doctor as to whether he is quite sure the nurse will be kind and not rough. The very idea is so repellent that indignation may be shown by the doctor, but it must not be shown by the nurse."

It is hardly necessary to particularise among the various contributions to a volume in which so high a level is maintained throughout. Dr. Bedford Pierce was fortunate in securing such a galaxy of talent, and by no means the least interesting sections are those for which he himself is responsible. In an historical introduction on "Pinel and Tuke," he describes the inception of the humane treatment of the insane, while in "Character and Outlook" he discusses, *inter alia*, the various theories of the causation of insanity. A mere list of the names of some of the contributors is a sufficient indication that the contents will be interesting. In addition to those already mentioned there are Dr. Bevan Lewis, Sir Thomas Clouston, Dr. Mercier, Dr. Percy Smith, Dr. Hubert Bond, Sir John Macpherson, Sir Robert Armstrong-Jones, and others. Without being unduly sententious and without setting forth ideals impossible of achievement, the lecturers have embodied in their addresses a vast amount of information and have laid down a high standard of ethics. As the editor rightly says, "The lectures represent the considered judgment of men with special knowledge of mental infirmity, and were it possible to spread abroad widely the lessons they contain, greater harmony would be promoted in social and family life." It is to be hoped that it will circulate far beyond the circle of workers in mental hospitals, and in due course become one of the classics of psychiatry.

### VERY WRONG FOOD.

**Right Food—The Right Remedy.** By Charles C. Froude, B.Sc. (Methuen and Co. 7s. 6d. net.)

Mr. Froude deals largely with the dangers of overeating and over-stimulating the body, and has succeeded well in pointing out the evil consequences. He is the first author, so far as we know, who has dealt in a detailed manner with the compatibilities and incompatibilities of our foods. Some of these have long been known, and are well known. But he has given long lists of what may be eaten together, and what should not be eaten together. Whether these are all incompatible is not obvious. He is somewhat contradictory. Thus he says:—"Meat should not be allowed with bread or other cereal foods because they are incompatible." Yet later on he says:—"Meat, fowl, game, fish, eggs, nuts and cheese do not combine with one another, nor with bread, cereals, or legumes; but," he continues, "there is no chemical incompatibility in combining these foods, but over-eating is the invariable result."

Mr. Froude advocates "no breakfast." In reply to this let us give a little evidence. We have made inquiries at several institutions where "the hands" formerly worked daily for two hours before breakfast; they then had their breakfast and continued to work until 12.30 for the next meal; both sexes were employed. After years of endeavour on the part of Union labour leaders the "no breakfast" rule was abolished. The hands now have breakfast before they come to work. Instead of working from 6 a.m. to 12.30 p.m., they work from 8 a.m. to 12.30 p.m. It is admitted on all sides that the effect on their work is very good; there has been a marked improvement in its quality and quantity. By 12.30 p.m. it is advanced nearly as far as when they began to work at 6 a.m. without breakfast, a clear saving of nearly two hours' time daily, with advantages to both manufacturer and worker.

The book is written on a distinct phase of dietism which knows little of scientific dietetics. It recommends the consumption of huge quantities of raw vegetables, especially of cabbages, carrots, parsnips, etc., in the form of a salad to be eaten at a mid-day meal. The quantity mentioned for consumption by one person is a vegetable dish full! But men and women are not sheep; moreover, how many people are able to eat raw carrots? One of the chief characteristics of the book is the excessive amount of raw food recommended. Mr. Froude applies the term "Acidosis" to very many conditions of the body; the word is used thirty-two times in less than a hundred pages. A strong protest must be made against such misuse and misapplication of a scientific word. He has no right to twist its meaning to suit his own ideas. We are not one-half of us suffering from diabetes, which we should be if we had genuine "Acidosis"; neither are we nearly all suffering from a mild degree of scurvy, which he considers is the cause of the "Acidosis."

### THE SICK CHILD.

**Pediatric Nursing:** Its Principles and Practice. By Bessie Ingersoll Cutler, R.N. (The Macmillan Co. 14s. net.)

The author of this book, who is a graduate of Massachusetts General Hospital Training School for Nurses, instructor in pediatric nursing, and supervisor of the Pediatric Department in the University of Minnesota, Minneapolis, besides having held many other posts in the world of sick children's nursing, is thoroughly conversant with her subject. She has produced a pleasantly-written book on the nursing care of sick children, which is written primarily for student nurses and is intended to be used in conjunction with lectures on the diseases of children. In all, there are twenty-five chapters, some dealing with the new-born and the premature infant, some with the growth and development of the normal child. A large section of the book is devoted to the subject of nutrition, breast-feeding occupying the first place as the "keystone of propaganda for the prevention of infant mortality and for the foundation of good health."

Artificial feeding is fully treated, trustworthy recipes and useful formulæ for times and quantities of food in sickness and health being added. The general medical and surgical nursing of children is detailed, and there is a special chapter on orthopædic care. The illustrations are numerous and excellent, and the book, which is well printed and strongly bound, will stand long service as a valuable work of reference on children's nursing.

### AN ENTERTAINING COLLECTION.

**A Whiff of Old Times.** Collected by John Wishart, M.D., D.Sc., Ch.B., F.L.S. (John Wright and Sons, Ltd., Bristol. 3s. net.)

It is not often that anyone benefits by a fractured clavicle, but, after reading Mr. Wishart's collection of "one hundred extracts from literature prior to 1850," we cannot help feeling that the enforced leisure which led him to make this collection was a piece of good fortune for his readers.

The book is written "For Medical Practitioners and Others." Medical practitioners will certainly be glad to have escaped the good old days when they read of 1841: "Luckless is he whom hard fates urge on To practise as a Country Surgeon, To drag a weary, galling chain, The slave of all for paltry gain, . . . For days and nights in some lone cottage, Condemn'd to live on crusts and pottage." Possible patients, moreover, will be glad to have escaped them when they hear of the country apothecary who "carries fate and physic in his eye; A potent quack, long vers'd in human ills, Who first insults the victim whom he kills," and of another, that, "He knew what's what, and that's as high As metaphysic wit can fly." In 1793 we find the complaint that, "For money doctors medicines compose, Bring you to life and bring it to a close. Many their conscience have for money sold." The collector is able to present to us, from the safe distance of nearly a century and a-half, extracts of rhyme and prose which, if written to-day, would arouse storms of indignation and are, happily, no longer true. "Others" will no doubt find entertainment both in the extracts containing allusions to medical men and matters and in those which have no other link of connection than that they appealed to Mr. Wishart's fancy.

### MISCELLANEOUS NOTICES.

### Mother and Child.

The Expectant Mother and Baby's First Month. By F. Truby King, C.M.G., M.B. (Macmillan. 2s. net.)—The first edition of this book was published some five years ago under the auspices of the New Zealand Government, and the present edition is practically a new book. It has been much enlarged, several new sections have been added, and is profusely illustrated. Dr. Truby King is so well known that his book needs no commendation, and its moderate price should make it easily accessible to those mothers to whom it will be especially valuable.

### Tips and Aphorisms.

Clinical Memoranda for General Practitioners. By Alec Theodore Brand, M.D., C.M., V.D., and J. R. Keith, M.A., M.D., C.M. Second Edition. (Baillière, Tindall and Cox. 7s. 6d. net.)—The subject-matter of this useful handbook has been rearranged according to the various systems of the body. A number of new diagnostic helps and fresh physical signs have been incorporated in the present edition, to which there has also been appended a copious index. The busy practitioner who turns to this book in any odd moment will be certain of finding within its pages something that will have a practical bearing upon his cases and shed new light upon some difficult point.

### Girth Control.

The Culture of the Abdomen: the Cure of Obesity and Constipation. By F. A. Hornibrook. With a Preface by Sir W. Arbuthnot Lane, Bt. (Heinemann. 6s. net.)—The author of this book, a life-long athlete, realised that he was putting on weight in an undesirable fashion, and he here relates his experiences in devising a new system of physical exercise whereby it is "possible to localise effort to the abdominal region, to promote more internal abdominal activity, and to concentrate on correct posture." Mr. Hornibrook's methods are entirely natural and are anatomically sound; moreover, they have been put to the test by many medical men with success. Briefly, the system consists of a series of movements designed to tone up the abdominal muscles and to prevent the "sagging" of the abdominal wall resulting in some degree of that enteroptosis which is all too common in people of both sexes. For sufferers from certain forms of chronic constipation, dyspepsia and obesity, as well as for ex-athletes who in middle age tend to develop a "corporation," the method described appears to offer the advantages of simplicity, suitability to age and sex, and last but not least, efficiency. As Sir Arbuthnot Lane remarks in his preface, it cannot fail to prove of the greatest service to the public, and also, we may say, to the medical profession as the natural educators of the public in health matters.

